# Volume Management in Hemodialysis—A Moving Target

**DOI:** 10.34067/KID.0000000000000108

**Published:** 2023-03-24

**Authors:** Simon J. Davies

**Affiliations:** School of Medicine, Keele University, Keele, United Kingdom

**Keywords:** BP, fatigue, person-centered care, intradialytic hypotension, bioimpedance, relative blood volume monitoring, lung ultrasound, left ventricular hypertrophy

## Introduction

My first exposure to hemodialysis was in 1976 when, as a first-year medical student, I visited the home of a friend with kidney failure. His bedroom was transformed into a mini hospital where he made up his own Kiil dialysers, used two independent volumetric pumps to control the blood flow, which he had learned to balance since the age of 16 years, and a weigh bed to monitor his intradialytic fluid removal. It left an impression. A lot has changed since then including the case mix of people on dialysis, their expectations and priorities, treatment goals and strategies, and technological advances. This brief article will look at how these have evolved over time and speculate as to how they might continue to develop (Table 1).

**Table 1:** Summary of past, present, and future states of volume management

Domain	Past	Present	Future
Case mix	Younger, less multimorbid population, able to tolerate large volume losses, typically hypertensive	Older, multimorbid population, frequently hypotensive and with more complex fluid distribution challenges and a stiff arterial circulation	Increasing proportion of old, frail, and muscle wasted multimorbid population in high-income settings and a global epidemic of diabetes
Inclusion of patient's priorities	Disease-centered approach—focused on laboratory measures and normalization of objective measures (*e.g.*, BP, left ventricular mass, Hb) One-size-fits-all approach that often did not take the patient perspective into account	Patient-centered care recognized and measures of important outcomes, *e.g.*, intradialytic hypotension and postdialysis recovery included in treatment goals	Person-centered care. Coproduction of treatment plans and prioritization of patient-reported outcomes, *e.g.*, fatigue, postdialysis recovery
Problem recognition	Prescription dominated by solute clearance Little attention to residual kidney function	Recognition that volume status (overhydration) is as, or more important than solute clearance, that high ultrafiltration rates are harmful, and both fluid balance and fluid distribution are critical	Volume management as a complex intervention that requires risk stratification. Requires validation of tools to support this approach and an evidence base
Treatment strategies	Prescription often designed to shorten hours maximizing solute clearance and divining target weight by using volume management to control BP	More frequent dialysis and avoidance of excessive fluid gains and methods to avoid intradialytic hypotension (Na modeling, dialysate temperature)	Negotiated (*e.g.*, goal directed) personalized approach, *i.e.*, risk-based (*e.g.*, the 3-d break increased mortality), and takes residual kidney function into account (*e.g.*, incremental start to dialysis)
Role of technologies	Focus on dialysis machine technology and delivery benefits *e.g.*, bicarbonate dialysate, volumetric ultrafiltration, erythropoietin, dialysate temperature regulation, and hemodiafiltration	Growing use of bioimpedance, lung ultrasound, and intradialytic relative blood volume monitoring which help identify problems but are yet to be demonstrated to add value in management of fluid status	Volume assessment tools will find their place as a component in the individualization of care Real-time intelligent feedback devices Easy-to-use home dialysis machines and wearable technologies

## A Changing Demographic

Since the early years of dialysis there has, at least in high-income countries, been a huge change in the demographic of the dialysis population, increasingly characterized by older people with increasing numbers of comorbid conditions. For a treatment that entails repeated episodes of cardiovascular stress, this represents a very real challenge, especially when it comes to volume management. For example, many of the factors that exacerbate intradialytic hypotension, the most commonly reported and troublesome dialysis complication, can be attributed to an aging and damaged cardiovasular system.^1^ Whereas hypertension was once the main concern of volume control, hypotension is now a significant risk factor and challenge when fluid needs to be removed. Fatigue, the symptom that hemodialysis patients would most wish to be rid of,^2^ is undoubtedly exacerbated by the increased frailty of this population, as is prolonged postdialysis recovery time, itself associated with worse survival.^3^

## Changing Expectations

Alongside these demographic changes have come different expectations. This is in part because of a change in the balance of importance between quality and quantity of life that naturally happens with aging but more fundamentally, a change in the nature of the relationship between health care professionals and the people they treat. We have moved from a disease-centered model of care through patient-centered and increasingly a person-centered care model, although full realization of this is still some way off.^4^ This has direct implications for volume management which hinges around the setting of the postdialysis target weight. By any definition, the setting of the target weight is a complex decision that needs to take many factors into account that influence both fluid balance and fluid distribution (see Table 2), but without coproduction of an agreed target with the patient, that may well require some negotiation, it cannot be claimed that person-centered care is happening.

**Table 2:** Categorizing the challenge of volume management by fluid balance versus fluid distribution

Fluid Balance Predominates	Fluid Distribution Predominates
**Positive balance**	
• Excessive salt (and water) intake	• Muscle wasted, frailty
• Large intradialytic weight gains	• Diabetic/autonomic neuropathy
• Requiring high ultrafiltration rates (*e.g.*, >10–13 ml/h per kilogram)• Anuria	• Cardiac dysfunction•Reduced ejection fraction: risk of pulmonary edema•Preserved ejection fraction: may require higher filling pressures to avoid hypotension
**Negative balance**	
• Inadequate fluid and nutritional intake	• Inflammation
• Diarrhea or vomiting	• Hypoalbuminemia
• High urine volume	• Sepsis
• Acute blood loss	

## An Evolving Strategy

For far too long, dialysis prescription was dominated by small solute clearance. This was in part driven by a desire to minimize treatment times but also a rather simplistic approach to fluid management, which went along the lines of “remove as much fluid as you can, preferably so that BP is controlled by volume management alone.” The focus on maximizing rapid solute clearance meant that volume management was often ignored, or worse, by forcing fluid removal into shorter and shorter dialysis treatments, it became positively detrimental. Although this might seem to belong to the distant past, a relatively recent survey of dialysis practices in the United Kingdom indicated that this was a common approach in half of the responding centers.^5^ The realization that high ultrafiltration rates, with their risk of intradialytic cardiac stress, hypotension, and organ damage, and that hypovolemia induced interdialytic thirst are all consequences of this approach has led to the recognition that more sophisticated strategies are needed. These concerns prompted the proposal for a volume first approach when prescribing dialysis, with consensus that volume status should be prioritized in clinical management, ultrafiltration rates limited, intradialytic sodium loading avoided, dialysate sodium concentration set between the range of 124–138 mEq/L, and dietary counseling should limit salt intake.^6^

More controversial is the choice of surrogate clinical outcomes to use when assessing the quality of volume management. Undoubtedly, left ventricular hypertrophy is a consequence of long-standing hypertension and fluid overload and thus a legitimate target for optimal fluid management. There is, however, poor evidence that reducing left ventricular mass translates into clinical benefit^7^ whereas medical treatment of hypertension is associated with better outcomes.^8^ It is also an example of a disease-centered approach to managing fluid status that runs the risk of putting one particular consequence of fluid overload, albeit a very important one, ahead of outcomes that might be even more important to patients. Daily dialysis might be good for left ventricular mass but comes at a treatment burden cost. Equally, to maximally protect the heart or other organs from hypertensive damage or fluid excess (*e.g.*, pulmonary edema), the optimal strategy may be to set the postdialytic weight below the normally hydrated weight, that is, cause volume depletion, whereas when protecting against dialysis-related symptoms, the opposite strategy must be adopted, with its inherent risks.

Perhaps most importantly, it is now recognized that preservation of residual kidney function is of value to hemodialysis patients,^9^ yet it is infrequently measured and rarely considered when prescribing dialysis. This is remarkable given the survival and quality-of-life benefits associated with residual kidney function, long appreciated in peritoneal dialysis patients. Aggressive fluid management strategies that do not take residual function into account run the risk of accelerating its loss, and there is an urgent need for trials of fluid management strategies that might preserve it for as long as possible. Conversely, there are people on dialysis who are at risk of dying during the 3-day break, and it is likely that at least some of those are at risk because of fluid excess, either directly because of pulmonary edema or indirectly by cardiac damage. It is clear that a one-size-fits-all strategy for volume management cannot deliver what is needed.

## The Role of Technologies

The single most important technological advance in volume management was the advent of volumetric ultrafiltration. The combination of a balanced pump system, a pair of pumps separated by a flexible membrane so that the dialysate flow in and out of the dialyser can be perfectly matched, with the addition of a volumetric ultrafiltration pump meant that fluid removal during dialysis could be controlled precisely. Its introduction meant that the rate of fluid removal could now be controlled, which previously was performed by monitoring weight change in response to positioning of the dialysis membrane. It had a major effect on the safety and symptoms associated with dialysis but also contributed to the drive to shorten dialysis treatments by maximizing ultrafiltration rates. The replacement of acetate with bicarbonate as the dialysate buffer also had significant benefits on intradialytic cardiovascular stability during dialysis of particular value in an increasingly comorbid population. Acetate infusion causes a drop in BP and is also associated with nausea on dialysis.

Both of these innovations became well established in the 1980s, whereas later technologies designed to help with volume management have been less universally adopted. These include hemodiafiltration, reported to reduce the incidence of intradialytic hypotension and improved survival—although still the subject of major trials in Europe—and devices designed to help with fluid management. These are intended to give the clinician insight into the fluid status and so provide support in the setting of the target weight. They include intradialytic relative blood volume monitoring, bioimpedance technologies, and other methods of measuring extravascular tissue fluid accumulation such as lung ultrasound. Relative blood volume monitoring exploits the idea that removal of fluid from the circulation by ultrafiltration will increase the hematocrit unless it is matched by plasma refilling from the interstitial extravascular fluid compartment. In theory, at least, once the refilling stops catching up with the ultrafiltration then the patient is at their target weight, and pattern recognition of the relative blood volume curve can be useful. Bioimpedance exploits the fact that when an alternating current is passed through tissues, it will meet less resistance from overhydrated interstitium but will be impeded by tissues whose cell membranes act as mini capacitors. It can be used to estimate the total body water and the proportion of this that is intracellular-versus extracellular. Lung ultrasound uses comets to detect extravascular lung fluid, so identifying subclinical pulmonary edema.

What all these methods share is an ability to identify patients at risk, and it is clear that tissue fluid excess, often subclinical, is associated with worse outcomes in hemodialysis patients. This seems to be independent of inflammation and BP but is as much a function of abnormal fluid distribution as it is of positive fluid balance (see Table 2), a feature of many chronic diseases that are associated with protein energy wasting. Indeed, loss of muscle mass of whatever cause, including deliberate or poverty-associated starvation, does not lead to a proportional reduction in the extracellular fluid volume.^10^ As clinicians, we can influence fluid balance through the dialysis prescription, but changing how fluid is distributed in the body is far more challenging. Trials evaluating these methods as useful guides to fluid management, including relative blood volume monitoring,^1^ bioimpedance,^11^ and lung ultrasound have not been promising overall.^12^ As such, these technologies should only be used in conjunction with a holistic clinical fluid assessment.^10^

## Looking to the Future

Given these limitations, changes in case mix and need to focus on the experience of dialysis, where are we going with volume management?^13^ The answer, surely, must be by taking a risk stratification approach that can accommodate individualized patient goals. This requires accurate diagnosis of the problem—for example, is it a fluid balance or fluid distribution problem (or both, Table 2), and a systematic approach to holistic fluid management that is underpinned by evidence. This requires the testing of approaches tailored to different risk groups through the conduct of robust trials, which despite being difficult to undertake are much needed. Technologies should steer away from nonstratified absolute target weight goals, be tested in different risk groups, and then develop artificial intelligence ultrafiltration profiling and/or feedback methodologies that are more likely to work successfully. Dialysis facilities need to find ways of designing their services so that flexibility can be accommodated—alternate day sessions for some patients, minimal care walk-in sessions that enable fluid management on demand or incremental dialysis for those with varying need or significant, and documented residual kidney function. Finally, easy-to-use home dialysis machines and the growth of successful home hemodialysis programs are needed.
